# Recent Advances in Gene Mining and Hormonal Mechanism for Brown Planthopper Resistance in Rice

**DOI:** 10.3390/ijms252312965

**Published:** 2024-12-02

**Authors:** Xiao Zhang, Dongfang Gu, Daoming Liu, Muhammad Ahmad Hassan, Cao Yu, Xiangzhi Wu, Shijie Huang, Shiquan Bian, Pengcheng Wei, Juan Li

**Affiliations:** 1 Key Laboratory of Rice Germplasm Innovation and Molecular Improvement of Anhui Province, Rice Research Institute, Anhui Academy of Agricultural Sciences, Hefei 230031, China; gudongfang65@aaas.org.cn (D.G.); ahmaduaf93@stu.ahau.edu.cn (M.A.H.); bianshiquan@aaas.org.cn (S.B.); 2 National Center of Technology Innovation for Saline-Alkali Tolerant Rice in Sanya, Sanya 572024, China; liudm0924@163.com; 3 State Key Laboratory of Hybrid Rice, Hunan Hybrid Rice Research Center, Changsha 410125, China; 4 College of Agronomy, Anhui Agricultural University, Hefei 230036, China; yc247178@163.com (C.Y.); lwxz21@stu.ahau.edu.cn (X.W.); 18326009151@163.com (S.H.); weipengcheng@gmail.com (P.W.)

**Keywords:** rice, brown planthopper, resistance gene, mechanism, phytohormone

## Abstract

Rice (*Oryza sativa* L.) feeds half the world’s population and serves as one of the most vital staple food crops globally. The brown planthopper (BPH, *Nilaparvata lugens* Stål), a major piercing–sucking herbivore specific to rice, accounts for large yield losses annually in rice-growing areas. Developing rice varieties with host resistance has been acknowledged as the most effective and economical approach for BPH control. Accordingly, the foremost step is to identify BPH resistance genes and elucidate the resistance mechanism of rice. More than 70 BPH resistance genes/QTLs with wide distributions on nine chromosomes have been identified from rice and wild relatives. Among them, 17 BPH resistance genes were successfully cloned and principally encoded coiled-coil nucleotide-binding leucine-rich repeat (CC-NB-LRR) protein and lectin receptor kinase (LecRK), as well as proteins containing a B3 DNA-binding domain, leucine-rich repeat domain (LRD) and short consensus repeat (SCR) domain. Multiple mechanisms contribute to rice resistance against BPH attack, including transcription factors, physical barriers, phytohormones, defense metabolites and exocytosis pathways. Plant hormones, including jasmonic acid (JA), salicylic acid (SA), ethylene (ET), abscisic acid (ABA), gibberellins (GAs), cytokinins (CKs), brassinosteroids (BRs) and indoleacetic-3-acid (IAA), play crucial roles in coordinating rice defense responses to the BPH. Here, we summarize some recent advances in the genetic mapping, cloning and biochemical mechanisms of BPH resistance genes. We also review the latest studies on our understanding of the function and crosstalk of phytohormones in the rice immune network against BPHs. Further directions for rice BPH resistance studies and management are also proposed.

## 1. Introduction

Rice (*Oryza sativa* L.) is a hydrophytic plant that is widely cultivated and feeds more than 50% of the world’s population and 60% of the Chinese population [1]. The brown planthopper (BPH, *Nilaparvata lugens* Stål) is considered one of the most devastating pests in rice-growing areas after the Green Revolution in the 1960s [2,3]. As monophagous and phloem-piercing herbivores, BPHs not only directly penetrate the rice sheath through the stylet and consume plant nutrients in the phloem but also cause indirect viral damage by transmitting rice viruses, including grassy stunt viruses and ragged stunt viruses. In addition, the excrement of BPH honeydew is beneficial for the proliferation of bacteria in rice. Heavy infestation by high-density BPH populations leads to a phenomenon called “hopperburn”, characterized by wilting, drying, dying and a considerable reduction in rice production [4]. Therefore, BPHs have been a main constraint to rice production, causing 20% to 80% yield loss and an economic loss of hundreds of millions of dollars globally almost yearly [5].

In current agricultural practices, the prevention and management of BPHs are primarily dependent on chemical insecticides at the cost of expense, labor force, human health and environmental protection. Additionally, BPHs develop more destructive biotypes with intensive virulence and pesticide tolerance due to the excessive application of pesticides, which subsequently results in more BPH outbreaks with greater lethality [6,7]. The long-distance migration of BPHs for better habitats and hosts further increases the difficulty of BPH management [8]. Thus, utilizing intrinsic host resistance, exploring rice-derived BPH resistance genes, elucidating the mechanism of rice defense against BPHs and planting resistant varieties are well recognized as the most effective, health-conscious and environmentally friendly strategies for BPH control [9,10].

In this review, we summarize the latest advances in studies on BPH resistance genes in rice and emphasize the genetic identification, cloning and functional analysis of these genes. Furthermore, this work introduces recent progress in elucidating the regulatory effects of plant hormones, an indispensable component of the rice immune network, on rice defense responses against BPHs, which have not been systematically discussed before. This review aims to deepen our understanding of rice–BPH interactions and provide important guidance for strategies for effective BPH management.

## 2. Evaluation of Rice Resistance to BPH

Given the unpredictability of insect behaviors and the fact that the occurrence of pest attacks is lightly subject to environmental factors, a series of techniques with high accuracy, efficiency and operability assume fundamental importance for estimating the resistance level of rice and screening resistant germplasms. To date, bulked seedling tests, standard seedbox screening tests (SSSTs), modified seedbox screening tests (MSSTs), tiller box screening techniques and field screening of cultivars have been developed to assess the reactions of host rice under BPH infestation [11,12]. With the development of screening methods, an increasing number of BPH resistance genes/QTLs have been identified and cloned [3,10]. The mass screening method of SSSTs has been used to evaluate the resistance of rice to BPHs at the juvenile stage, generally at the two- or three-leaf stage, with second-instar BPH nymphs. However, the SSST is also subject to various environmental and developmental factors including temperature, humidity, biotype, nymph stage and predators of BPHs, and setting up more experimental replications and scenarios may increase the accuracy of BPH resistance assessment. Even though the SSST is recognized as the standard and mainstream method for phenotyping rice BPH resistance due to its convenience and time-saving characteristics, it is widely utilized for rapid, high-throughput identification of resistant resources and genes.

BPH’s feeding on rice is a mutually influential process; we can indirectly measure rice’s resistance/susceptibility level by probing the physiological and biochemical reactions of rice plants to BPHs. These methods include the host choice test, honeydew excretion test, fecundity test, mortality of nymphs and electronic penetration graph (EPG) technique [13,14]. In general, these methods evaluate the resistance level of rice through the settling, feeding, development, fecundity and survival of BPH influenced by rice, which will enable us to excavate minor BPH resistance genes/QTLs and dissect the resistance mechanism in rice. The resistance of host plants to insects can be physiologically categorized into three mechanisms: antixenosis, antibiosis and tolerance [15]. Antixenosis influences insects’ preference, settling and oviposition, whereas antibiosis impedes the feeding, growth and survival of insects on hosts [16]. Thus, these methods can be used to analyze the resistance mechanism of rice to BPHs. In short, crossvalidation through a combination of several methods and approaching with enough caution are key to evaluating rice resistance to BPHs.

## 3. Screening of BPH-Resistant Rice Germplasms

**Figure 1:**
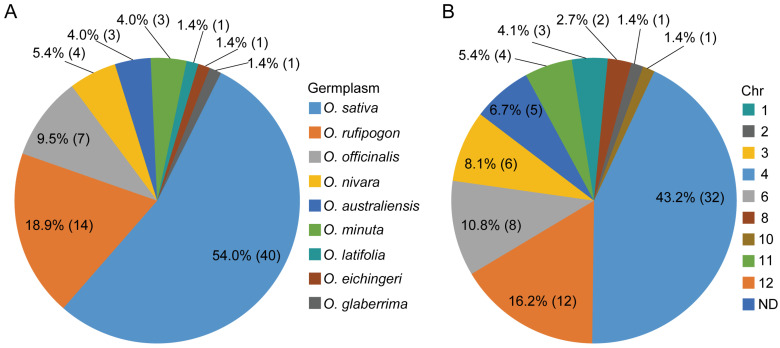
Distribution of BPH resistance genes/QTLs in rice chromosomes and germplasm resources. Numbers in parentheses represent the number of BPH resistance genes/QTLs in corresponding chromosomes (**A**) and germplasm resources (**B**).

Excavation of rice germplasms that confer resistance to BPHs is the initial step for studying the resistance of rice plants to BPHs and breeding resistant varieties. The identification of BPH-resistant rice germplasms dates back to 1969, when Mudgo, the first rice resource with BPH resistance, was reported by the International Rice Research Institute (IRRI) [17]. Numerous BPH-resistant rice resources have subsequently been screened and identified from cultivated and wild rice varieties, e.g., ASD7, Rathu Heenati, Babawee, Swarnalata, T12 and Balamawee [18,19]. Overall, *indica* cultivars are more resistant to BPHs than *japonica* subspecies. Furthermore, rice germplasms with BPH resistance are abundant in wild rice species (Figure 1A). Possibly because of the lower selective pressure, greater genetic variation and hostile living environment of wild rice than those of cultivated varieties [19,20]. For example, *Bph27* and *Bph29* derived from *Oryza rufipogon* and *Bph13*–*Bph16* were identified in *Oryza officinalis*, and several BPH resistance genes were also discovered in Oryza *australiensis*, *Oryza minuta*, *Oryza glaberrima* and *Oryza eichingeri* [3,14,21]. However, only a few germplasms confer resistance to all BPH biotypes, which raises the risk of the breakdown of BPH resistance by new dominant biotypes. Thus, exploring and identifying more germplasms with broad-spectrum and durable BPH resistance is crucial and urgent.

## 4. Mapping of BPH Resistance Genes/QTLs in Rice

In 1971, *Bph1* and *Bph2* were first identified by the IRRI from Mudgo and ASD7, respectively, and were finally mapped to chromosome 12 [22,23,24,25]. To date, 74 BPH resistance genes/quantitative trait loci (QTLs) showing different responses to different BPH biotypes have been identified in rice from various gene pools through different genetic populations and most of these loci have been mapped to chromosomes (Figure 1, Table 1). To eliminate the disorder caused by the duplication of names for BPH resistance genes, a chronological ranking pattern was adopted in this study. Approximately half of these genes were detected in cultivated rice varieties, and the other half were derived from wild rice species, including bph18(t), bph19(t)-2, bph20(t), bph21(t), bph22(t), bph23(t), bph24(t), Bph27, Bph29, Bph35, Bph36, Bph38, Bph41-1 and bph42 from *O. rufipogon*; bph11, Bph14, Bph13(t)-2, Bph15, Bph16, qBph3 and qBph4 from *O. officinalis*; Bph34, bph39(t), bph40(t) and Bph45 from *O. nivara*; Bph10, Bph18 and qBph4.2-1 from O. australiensis; Bph20(t), Bph21 and Bph23(t) from *O. minuta*; Bph12 from *O. latifolia*; Bph13(t)-1 from *O. eichingeri*; and Bph22(t) from *O. glaberrima* (Figure 1A). Notably, the wild rice group forms a plentiful pool for screening and isolating BPH resistance genes.

**Figure 2:**
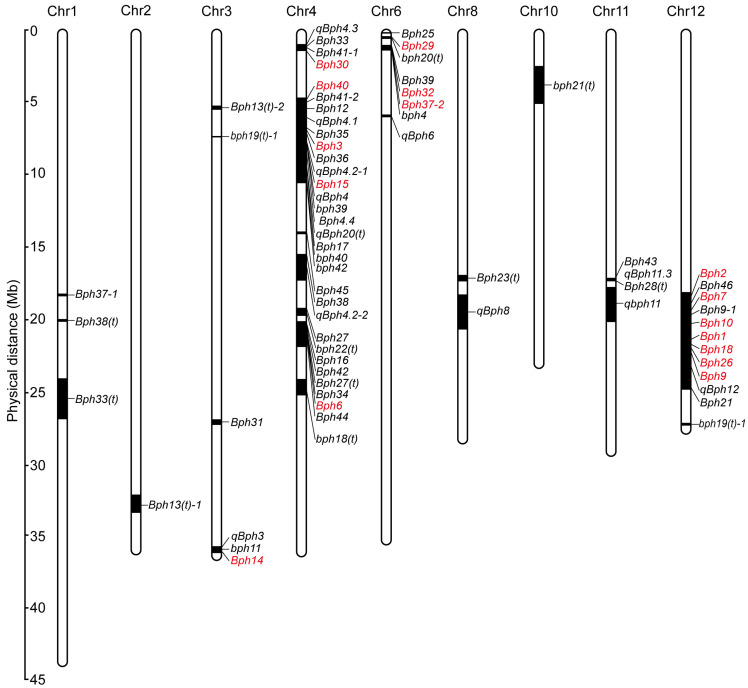
Chromosomal locations of BPH resistance genes/QTLs in rice. A total of 74 BPH resistance genes/QTLs have been identified in rice. Among them, except *bph5*, *bph8*, *Bph22(t)*, *Bph23(t)* and *bph24(t)*, 69 genes/QTLs have been mapped on 9 chromosomes. Numbers on the left indicate the physical distance. Black bars represent the genetic region of BPH resistance genes/QTLs. Red and black represent genes that have been cloned and genes that have not been cloned, respectively.

These BPH resistance genes are distributed on nine rice chromosomes, and most are clustered on chromosomes 3, 4, 6 and 12. Four clusters, which occupy 52 genes or QTLs, have been reported on the long arm of chromosome 12, the short arm of chromosome 6 and the short and long arms of chromosome 4, indicating a convergent effect may exist in the evolution of BPH resistance genes (Figure 1B and Figure 2). These genetically close genes in the same cluster may represent different genes or alleles of one gene, considering the discrepancy in the genetic background of different BPH-resistant donors and the complexity of identifying BPH-resistant genes. Despite the confusion in designation, positional ambiguity and limited knowledge of some BPH resistance genes, these genes still provide numerous genetic resources for research on BPH resistance and the breeding of resistant rice cultivars.

## 5. Cloning and Mechanistic Analysis of BPH Resistance Genes in Rice

With great advances in genome sequencing techniques and molecular biology research methods, breakthroughs have been made in the cloning and functional analysis of BPH resistance genes (Table 2). *Bph14*, which encodes a coiled-coil nucleotide-binding leucine-rich repeat (CC-NB-LRR) protein, was the first identified and characterized BPH resistance gene through map-based cloning [11]. *Bph14* is strongly expressed in vascular bundles where the BPH feeds. The salicylic acid (SA), callose biosynthesis, reactive oxygen species (ROS) generation and trypsin inhibitor pathways are activated in plants harboring *Bph14* upon BPH infestation. In addition, the CC and/or NBS domains of Bph14 are sufficient to confer the resistance level of the full-length Bph14 to BPHs [86]. The homodimer of Bph14 can interact with the transcription factors WRKY46 and WRKY72 and protect them from degradation, thereby increasing the binding activity of WRKY46/72 to the promoters of a callose synthase gene and a receptor-like cytoplasmic kinase gene, ultimately eliciting a resistance response in rice.

A spectacular study revealed the molecular and cellular mechanisms involved in host plant resistance to herbivores through the functional identification of an effector for the receptor Bph14 [87]. BISP, a salivary protein from the BPH, is secreted in rice and blocks the basal defense response by interfering with the phosphorylation activity of OsRLCK185 in susceptible plants. In Bph14-containing plants, BPH14 directly recognizes BISP through the LRR domain and promotes resistance against BPHs. Furthermore, the BISP-BPH14 complex directly binds the selective autophagy cargo receptor OsNBR1 and induces the degradation of BISP in an autophagic manner. In turn, this fine-tuning of the rice resistance response protects plants from the adverse effects on growth and reproduction caused by the continuous activation of BPH14-mediated immunity. Since BISP was identified as the first salivary protein perceived by plant immune receptors and induces host defense, the gene-for-gene hypothesis between insects and hosts has been further verified [88].

*Bph26*, identified from the *indica* rice cultivar ADR52, also encodes a CC-NB-LRR protein [56]. *Bph26* is intensely expressed in vascular bundles of the leaf sheath and confers antibiosis to BPHs. Sequence comparison revealed that *Bph26* is identical to *Bph2*. *Bph26*-mediated resistance is broken down by biotype 2 of the BPH, so it is of low value to apply Bph26 in the production practice of rice breeding. *Bph18*, located on the long arm of chromosome 12, was cloned from the wild species O. australiensis [47]. *Bph18* encodes an atypical NBS-LRR protein with two NBS domains and is widely spread throughout the endomembrane system. *Bph18* is strongly expressed in the vascular bundles of the leaf sheath, indicating an inhibitory effect of *Bph18* on BPH feeding. Sequence and functional analyses revealed that *Bph18* and *Bph26* are functionally different alleles.

*Bph3* was identified from the Sri Lankan *indica* cultivar Rathu Heenati and conferred resistance to four BPH biotypes [89,90]. Although *Bph3* has been utilized in rice breeding for more than 40 years and has robust resistance to BPHs, the cloning of *Bph3* was not accomplished until 2014 [28,91]. First, *Bph3* exhibits strong antixenosis and antibiosis to BPHs; as a result, the feeding behavior and development of BPHs are severely dampened in plants containing *Bph3*. Further map-based cloning and functional characterization demonstrated that the *Bph3* locus is located on the short arm of chromosome 4 and consists of three lectin receptor kinase genes (OsLecRK1, OsLecRK2 and OsLecRK3). OsLecRK1 was previously reported as *Bph15* or OslecRK, which regulates rice seed germination [45]. Genetic and transgenic analyses revealed that more OsLecRK genes in *Bph3* locus pyramids resulted in more stable and broad-spectrum resistance to BPHs and WBPHs. OsLecRKs are located on the plasma membrane and are considered crucial receptors for perceiving herbivore-associated molecular patterns to trigger downstream defense signaling.

*Bph29*, a recessive BPH resistance gene, was isolated from the *indica* rice introgression line RBPH54, derived from wild rice *Oryza rufipogon* [51]. Wang et al. finely mapped *Bph29* to a 24 kb interval on the short arm of chromosome 6 and successfully cloned it [21]. *Bph29* encodes a nucleus-localized B3 DNA binding domain-containing protein. *Bph29* is markedly suppressed by BPH infestation and is specifically expressed in vascular tissue. Upon BPH attack, the SA pathway is stimulated, yet the jasmonic acid (JA) and ethylene (ET) pathways are inhibited by *Bph29*. Therefore, *Bph29* may coordinate with plant hormone pathways to increase rice resistance to BPHs.

*Bph32*, also mapped on the short arm of chromosome 6, was derived from the resistant rice variety Ptb33 [61]. BPHs’ survival rate and honeydew significantly decreased on plants introduced with *Bph32*, indicating that *Bph32* confers antibiosis to BPHs. The expression level of *Bph32* is strongly increased in the leaf sheath, parenchyma cells and vascular bundle, which further increases following BPH attack. *Bph32* encodes a special protein containing a short consensus repeat (SCR) domain and localizes to the plasma membrane. Sequence annotation revealed that *Bph32* in Ptb33 shares 100% sequence identity with its allele in *Oryza latifolia*, suggesting that *Bph32* may originate from wild rice.

*Bph9* was cloned from the long arm of chromosome 12 in the *indica* rice variety Pokkali and was shown to exert antixenotic and antibiotic effects on BPHs [36]. *Bph9* encodes a rare CC-NBS-NBS-LRR protein and is widely distributed throughout the endomembrane system. Functional assays revealed that *Bph9*-mediated resistance presumably depends on the induction of cell death and the activation of the SA and JA pathways. Further sequence analysis revealed that *Bph9* and seven other genes (*Bph1*, *Bph2*, *Bph7*, *Bph10*, *Bph18*, *Bph21* and *Bph26*) in the same locus are allelic genes to each other. These eight genes can be categorized into four allelotypes (*Bph1/9-1*, *-2*, *-7* and *-9*), which confer diverse resistance levels to different BPH biotypes. *Bph1/9-1* (*Bph1*, *Bph10*, *Bph18* and *Bph21*), the earliest allelotype, is used in rice breeding first and shows resistance to biotype 1 of the BPH. The allele *Bph9-2* includes *Bph2* and *Bph26* and is resistant to biotype 2 of the BPH. *Bph7* and *Bph9* belong to *Bph9-7* and *Bph9-9*, respectively, and both allelotypes confer high resistance to the major three BPH biotypes. The allelic variation in Bph9 empowers rice to confront the variation in BPH biotypes, revealing the coevolution between rice and BPHs and shedding new light on rice breeding for insect resistance.

Guo et al. successfully fine-mapped and cloned *Bph6*, which was previously mapped to the long arm of chromosome 4 and conferred antixenosis and antibiosis to BPHs from the Bangladesh landrace Swarnalata [33]. *Bph6* is highly expressed in vascular bundles, sclerenchyma tissues and companion cells. *Bph6* encodes an unidentified protein and colocalizes with the exocyst complex. *Bph6* interacts with the exocyst complex subunit OsEXO70E1 and increases exocytosis; the knockdown of OsExo70E1 also weakens the resistance level controlled by *Bph6* [32]. *Bph6* also interacts with OsEXO70H3 and S-adenosylmethionine synthetase-like protein (SAMSL) to facilitate lignin deposition for maintaining and reinforcing the cell wall in sheaths [92]. In addition, *Bph6* promotes a phytohormone pathway that integrates SA, JA and CK to increase BPH resistance. *Bph6* displays broad resistance to all biotypes of the BPH and WBPH; moreover, introgression with *Bph6* has no adverse influence on the growth and yield of rice plants [32].

*Bph30*, a completely dominant gene that confers antibiosis and antixenosis resistance to BPHs and WBPHs, is located on the short arm of chromosome 4 in landrace AC-1613. *Bph30* encodes a novel protein that consists of only two leucine-rich domains (LRDs). *Bph30* is highly expressed in the sclerenchyma cells of leaf sheaths. It facilitates hemicellulose and cellulose deposition to increase the hardness and thickness of the cell wall, ultimately acting as a mechanical barrier to impede the feeding of BPHs on rice [59]. Strikingly, this study revealed that smooth and rough areas are arranged regularly on the leaf sheath surface. BPHs prefer to penetrate smooth and long cell blocks with stylets to pass through sclerenchyma and suck phloem sap rather than rough blocks that are covered with papicles and glochids.

With the development of sequencing technology and the rise of bioinformatics, genome-wide association studies (GWASs) have become an efficient new method for screening and identifying BPH resistance genes in addition to map-based cloning. Shi et al. cloned *Bph40*, a homologous gene of *Bph30*, via a GWAS of 1350 rice varieties worldwide [68]. An SNP in the exon of *Bph40* divides these varieties into two categories, haplotypes A (wild type) and B (mutant type), and plants harboring haplotype B confer significantly greater resistance to BPH attack than those harboring haplotype A. Similarly, *Bph40*-mediated resistance against BPHs is also attributed to cell wall fortification in sclerenchyma. *Bph37*, encoding a CC-NB-LRR protein without an LRR domain on the short arm of chromosome 6 in the resistant accession SE382, was isolated via GWAS combined with map-based cloning [68].

The cloning and functional identification of BPH resistance genes facilitate the comprehensive analysis and deployment of rice defense responses against BPH infestation and deepen our understanding of the interactions between herbivores and host plants.

## 6. Roles of Phytohormones in BPH Resistance

**Figure 3:**
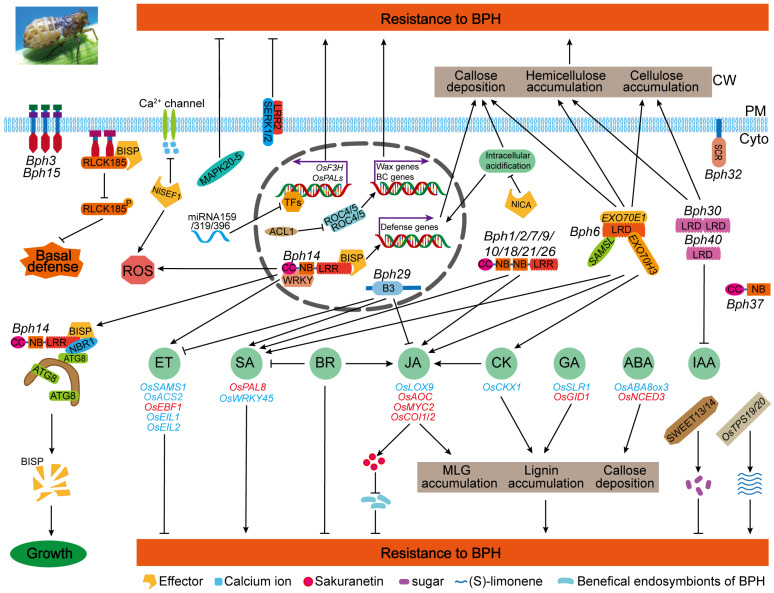
The preliminary model of rice defense system against BPHs. Multiple factors take part in rice resistance to BPHs, including transcription factors, phytohormones, mitogen-activated protein kinase cascades, calcium signaling, ROS, microRNAs, defense gene expression, post-transcriptional modification, metabolites pathways, etc. The phytohormones and cell wall thickening (accumulation of cellulose, lignin, callose, etc.) seem to be two of the key regulators in the BPH resistance system of rice. ↓ and ⊥, respectively, indicate the direct or indirect activation and inhibition effect on rice defense responses to BPHs. Genes written in red and blue, respectively, indicate that the genes confer positive and negative regulation on BPH resistance in rice with direct genetic evidence. ET, ethylene; SA, salicylic acid; BR, brassinosteroid; JA, jasmonic acid; CK, cytokinin; GA, gibberellin; ABA, abscisic acid; IAA, indole-3-acetic acid; TFs, transcription factors; BC, bulliform cell; ROS, reactive oxygen species; MLG, mixed-linkage b-1,3;1,4-D-glucan; PM, plasma membrane; Cyto, cytoplasm; CW, cell wall.

Rice exploits an integrated and sophisticated defense strategy to detect and resist attack from the BPHs, including physical barriers, the mitogen-activated protein kinase (MAPK) cascade, calcium signaling, ROS, transcription factors, microRNA, the metabolite pathway, intracellular pH modulation and post-transcriptional modifications [93,94,95,96,97,98,99,100,101,102] (Figure 3). Incontestably, phytohormones are indispensable components of a significant portion of rice’s immune system against BPHs.

SA modulates rice defense responses to BPHs as a typical piercing-sucking herbivorous insect. The SA pathway takes part in *Bph6*-, *Bph9*-, *Bph14*- and *Bph29*-mediated resistance of rice to BPHs [11,21,32,36]. The transcript levels of SA-related genes, such as *OsPAL*, *OsICS1*, *OsNH1* and *OsPR5*, and the SA content elevate after BPH infestation. Exogenous treatment with SA or increasing endogenous SA accumulation by overexpressing *OsPAL8* raises rice survival in response to BPH attack. Conversely, SA-deficient mutants resulting from ectopic expression of the bacterial salicylate hydroxylase gene (*NahG*) or knockdown of *OsPALs* display severely increased susceptibility to BPH infestation [32,98,103]. However, *OsWRKY45*, a key factor downstream of SA signaling, negatively regulates rice resistance to BPHs [104].

In consideration of the primary regulatory function of JA in defense against chewing herbivores and the antagonistic effect between SA and JA, JA was first regarded as a negative modulator of BPH resistance in rice [105,106]. In agreement with this view, *Bph14* and *Bph29* inhibit the expression of JA-related genes and JA accumulation [11,21]. The silencing of *OsHI-LOX*/*OsLOX9* impedes BPHs’ settling and ovipositing behaviors on rice plants [107]. However, with in-depth research and findings, JA seems to play a strong role in regulating the BPH resistance of rice. The expression levels of JA pathway genes and JA content in rice are induced by introgression from *Bph6* and *Bph9* or infestation with BPHs. Knockout of *OsAOC*, a JA biosynthesis-related gene, *OsMYC2*, a key transcription factor in JA signal transduction, and the JA receptors *OsCOI1* and *OsCOI2* promoted the susceptibility of rice to BPHs [108,109]. In addition, exogenous JA treatment enhances rice resistance to BPHs [32,36,110]. JA-Ile is generally recognized as the most bioactive JA derivative [111]. Nevertheless, hydroponic treatment with the JA–amino acid conjugates JA-Val or JA-Leu, but not JA-Ile, activates the JA pathway and increases the accumulation of the defensive compounds trypsin proteinase inhibitors and phenolamides, resulting in enhanced resistance to BPHs in rice [112]. JA also increases the accumulation of sakuranetin, a flavonoid phytoalexin, which protects rice by eliminating the beneficial endosymbionts of the BPHs [96]. Furthermore, the molecular mechanism underlying JA-mediated BPH resistance has been partly elucidated [113]. OsMYC2 directly targets the promoter of the glucan synthase gene *OsCslF6* to facilitate mixed-link β-1,3;1,4-D-glucan (MLG) biosynthesis and enhances BPH resistance by reinforcing vascular wall thickness. These findings suggest that SA and JA both positively contribute to resistance responses against BPH in rice.

In addition to SA and JA, other defense phytohormones, ethylene (ET) and abscisic acid (ABA), have also been reported to participate in rice resistance against BPHs. The ET pathway is repressed in plants expressing *Bph29*, and decreased emission of ET through silencing the biosynthesis gene *OsACS2* elevates the BPH resistance level in rice [21,114]. ET synergizes with the JA pathway via an OsEBF1-OsEIL1-OsLOX9 module to fine-tune rice responses after BPH infestation [115]. Additionally, the negative role of ET in the resistance of rice to BPHs accounts for the susceptibility induced by dim light in an *OsEIL2*-dependent manner [116]. ABA acts as a crucial plant hormone for biotic or abiotic stress, and the association between ABA and BPH resistance has also been preliminarily investigated. Overexpressing the synthase gene *OsNCED3* or silencing the hydrolase gene *OsABA8ox3* of ABA increases BPH resistance in rice [117,118]. Rice plants exogenously treated with ABA presented increased resistance to BPH feeding. ABA promotes callose deposition, including enhancing callose synthase activity and suppressing hydrolase β-1,3-glucanase activity, to modulate BPH resistance in rice [117,119]. Therefore, although both are crucial plant hormones for the rice response to stresses, ET and ABA play opposite roles in regulating rice resistance to BPHs.

Besides classic defense-related hormones, growth-related phytohormones constitute vital parts of the rice defense system against BPHs. Gibberellin (GA) functions as a positive regulator of BPH resistance. Overproduction of GA interferes with the performance of BPHs on rice plants, such as lower preference and higher nymph mortality [120]. The activation of GA signaling by the overexpression of the GA receptor *OsGID1* or the knockout of the DELLA protein-encoding gene *OsSLR1* and exogenous treatment with GA increase BPH resistance in rice, which may be attributed to the reprogramming of phytohormone pathways and increased lignin content [121,122,123]. Guo et al. reported that cytokinin (CK) coordinates with the SA and JA pathways to form an integrated phytohormone network involved in BPH resistance mediated by *Bph6* [32]. The transcript levels of CK-related genes and the CK content significantly increased upon BPH attack [32,110]. The increase in endogenous and exogenous CK promotes rice resistance to BPHs by facilitating the JA pathway and increasing lignin accumulation in the rice sheath. However, brassinosteroid (BR) is reported to negatively regulate BPH resistance by activating the JA pathway and repressing the SA pathway [103]. Similarly, indoleacetic acid (IAA) also plays a negative role in the BPH resistance of rice. The IAA level in resistant rice plants is substantially lower than that in susceptible rice after BPH infestation, and exogenous spraying with IAA decreases the tolerance of rice to BPHs [124,125].

## 7. Discussion and Future Perspectives

BPHs, one of the most devastating stressors for rice production, are currently controlled by massive application of chemical insecticides, inevitably at the cost of environmental pollution, human health and the immune system of rice. The identification and isolation of BPH resistance genes are fundamental and pivotal for breeding resistant rice cultivars and constitute the most practical and eco-friendly method for BPH management [28]. Although more than 70 genes/QTLs have been reported from rice and wild relatives, a noteworthy disorder problem still exists in naming BPH resistance genes. First, duplication is prevalent; for example, two *Bph37* and two *Bph41* genes located in different genetic regions have been reported [67,68,73,74]. Second, the use of the suffix ‘(t)’ represents ‘tentative’, and the prefix ‘q’ represents ‘QTL’, such as in the genes *Bph27* and *Bph27(t)* as well as *Bph6* and *qBph6*, further confusing the distinction of different genes [14,32,57,80]. These issues hinder the study and breeding exploitation of BPH resistance genes. The chronological ranking pattern we adopt in this study may lose efficacy when more resistance genes are reported in the near future. We think that a naming rule based on resistant donors, chromosomal locations and chronological order will be conducive to solving the problem. Therefore, rigorous and normative nomenclature criteria need to be established, which scholars in this field propose and promote together to advance the research and breeding application of BPH resistance efficiently and effectively.

The mechanism of rice BPH resistance has been scientifically elucidated with an integrated and effective defense network being depicted (Figure 3). Rice adopts a strategy similar to the defense against pathogens, including pattern-triggered immunity (PTI) and effector-triggered immunity (ETI) to resist BPH attacks. Accordingly, most cloned BPH resistance genes encode membrane receptor kinase and intracellular NB-LRR receptor (NLR) proteins [11,28]. In the ETI pathway, the recognition of effectors and downregulation of defense responses (such as ROS, autophagy, plant hormones, physical reinforcement and defense gene expression) have been well studied [36,59,87]. However, the activation of BPH-resistant NLRs remains unknown. NLRs form protein complexes called resistosomes via conformational changes to activate NLR immune responses [126]. Hence, structural elucidation is urgently needed on NLRs conferring BPH resistance, which will contribute to further understanding of the rice immunity mechanism against insects and promote breeding of insect resistance with NLR genes. Meanwhile, *Bph3* may act as a pattern recognition receptor (PRR), but the PTI-mediated BPH defense is poorly understood. The recent finding of OsLRR2, a plasma membrane-localized leucine-rich repeat protein, reveals PTI’s important participation in rice BPH resistance [127]. OsLRR2 impairs OsFLS2/OsPEPR1-mediated immunity by competitively interacting with co-receptors OsSERK1/OsSERK2, decreasing BPH resistance. However, the identification and recognition of BPH-associated molecular patterns (BAMPs), which will provide promising targets for developing insecticides for chemical control and are worthy of more attention, remain extremely scarce.

Phytohormones play irreplaceable roles in the rice defense system, and SA/JA are the two most studied plant hormones associated with BPH resistance. Although the positive regulatory effects of SA and JA on rice resistance against BPHs have been extensively investigated, their roles still seem somewhat obscure. In contrast, the JA pathway is repressed in plants expressing *Bph14* or *Bph29*, and the knockout of several JA-related genes results in increased resistance to BPHs [11,21]. Xu et al. reported that BPHs perform similarly on wild-type and SA-deficient plants [108]. Here, we propose three explanations for this paradoxical phenomenon. First, plant hormones are efficient and sensitive, and different concentrations could impose distinct effects on plants [128,129]. Mutations in a single gene individually may have a limited impact on the content and signaling of the entire multicomponent phytohormone pathway, resulting in different responses of rice to BPH infestation. Second, due to their mutual effects, modifications of target phytohormones may alter other hormone pathways and lead to discrepant or opposite outcomes in the BPH–rice interaction. As mentioned above, GA positively regulates BPH resistance [121]. However, the knockout of *GAMYBL2* facilitated GA biosynthesis and increased susceptibility to BPH infestation in rice, probably partly because of the concurrent promotion of BR signaling [130,131]. Third, different genetic backgrounds of plant materials, evaluation methods and criteria for phenotyping BPH resistance, including the developmental stages of plants and BPHs, could influence the identification and judgment of BPH resistance. For example, a recent study revealed that gravid BPHs and nymphal BPHs induce different responses in rice plants with mutations in the sugar transporter gene OsSUT2, including the length and number of BPH salivary sheath branches [132]. Thus, the results of the present study suggest that JA and SA play prominent positive roles in the resistance of rice to BPHs, and more crucial phytohormonal factors regulating BPH resistance should be identified as candidates for improving BPH resistance in rice.

The crosstalk between phytohormones plays a pivotal role in the plant immune system, but a two-sided role of this crosstalk has been presented in modulating rice resistance to BPHs. Plant hormones exploit antagonistic effects to maximize their own benefits and are generally accompanied by losses or compromises in other aspects; a well-known example is the growth–defense trade-off [133]. For instance, although both JA and GA positively affect rice resistance to BPHs, JA deploys MYC2-GA2ox/JAZ-DELLA modules to hijack the GA pathway and promote BPH resistance with restricted growth in rice [134]. Meanwhile, due to the versatility of phytohormones in regulating plant biological and physiological processes, the interactions between phytohormones are conducive to fine-tuning and optimizing resistance and growth in rice [135,136]. *Bph6*-containing rice plants activate an integrated defense network with the JA, SA and CK pathways and enhance BPH resistance without adversely influencing rice agronomic traits or grain yields [32]. Taken together, the elaboration of the hormonal roles in BPH resistance will provide valuable targets for breeding resistant cultivars against BPHs, and an integrative and synergetic phytohormone pathway adopted by rice is advantageous for tailoring defense responses against BPHs through the compatibility of high-level resistance and growth.

To date, investigations of BPH–rice interactions have focused on identifying dominant resistance (R) genes in host plants, especially wild rice. However, the divergent genetic background, linkage with adverse genes, lack of broad-spectrum resistance genes and emergence of new biotypes of the BPH, along with time-consuming and labor-intensive phenotyping and breeding work, deeply impede the isolation and application of BPH resistance genes. Accordingly, although more than 70 BPH resistance genes/QTLs have been identified, only 17 genes have been cloned and, among them, 8 are alleles. Several elite rice varieties resistant to BPHs have been developed by introducing BPH resistance genes, such as *Bph3*, *Bph6* and *Bph9*, via marker-assisted selection (MAS). However, considering the experience of *Bph1* and *Bph2*, more attention should be attached to the risk that currently applied resistance could be overcome by the adaptation of BPHs in the short term [28,137,138]. Effectiveness and sustainability should occupy equivalent position in BPH-resistant breeding. In general, plant susceptibility (S) genes are exploited by pathogens/insects through effectors to facilitate successful infection [139]. In this scenario, two alternatives can also be considered besides identifying more BPH-resistant R genes in rice. First, unlike resistance mediated by the R gene, S gene mutations can result in recessive, nonrace-specific, potentially durable, broad-spectrum resistance in plants [140]. For example, genetic manipulation of the S gene MLO enhances wheat resistance to powdery mildew without sacrificing growth or yield [141]. Several S genes regulating rice resistance against BPHs have recently been identified. Rice plants confer robust BPH resistance, and higher yields have been achieved by the knockout of OsLRR2 [127]. SWEET and bulliform cell-related genes also show great potential as candidate targets of the S genes for improving BPH resistance [100,142]. So, combining the screening and identification of BPH-susceptible genes with burgeoning genome-editing technology strikingly provides an effective and promising breeding strategy for rice resistant to BPHs. Second, during the arms race between the host and pathogen/insect, effectors and elicitors secreted from the latter into the former can suppress and activate plant’s defense response [143]. The discovery of the first effector BISP in BPHs shed new light on BPH management. Ectopic expression of *Bisp* increases the sensitivity of susceptible rice; however, it further enhances the tolerance of plants harboring immune receptor Bph14 to BPH infestation [87]. Recently, intracellular acidification, which could be repressed by a salivary carbonic anhydrase protein NICA in BPHs, has been proved as a previously uncharacterized defense mechanism against herbivores in rice [101]. As a result, using effector or elicitor genes from BPHs represents a new approach to pyramid rice resistance levels and preventing BPH attacks.

In summary, exploitation and introgression of resistance genes, which can be substantively used in breeding, remain the most effectively practicable and environmentally friendly management approach for BPH control. Moreover, further efforts are needed to elucidate the functions and connections of BPH resistance genes, clarify the regulatory network of plant hormones in BPH resistance and dissect the coevolution mechanism between rice and BPHs. These studies will deepen our comprehensive understanding of host–insect interactions, lay a foundation and provide new perspectives for crop improvement. In addition to traditional genetic and molecular biology techniques, biological control, high-throughput sequencing, gene editing technologies, synthetic biology, bioinformatics and artificial intelligence should play more important roles in this process.

## Figures and Tables

**Table 1:** BPH resistance genes/QTLs in rice.

*Genes/QTLs*	Chr.	Germplasm	Linked Markers	Position (Mb)	**Resistance Mechanism**	Resistance to Biotype	Reference
Bph1*	12L	Mudgo	pBPH4, pBPH14	22.86	Antibiosis, antixenosis	1, 3	[22,26]
Bph2*	12L	ASD7	RM463, RM7102	13.21–22.13	Antibiosis, tolerance	1, 2	[25,27]
Bph3*	4S	Rathu Heenati	RHD9, RHC10	6.20–6.97	Antibiosis, antixenosis	1, 2, 3, 4	[28]
*bph4*	6S	Babawee	RM589, RM586	1.38–1.47	Antibiosis, antixenosis	1, 2, 3, 4	[29,30]
*bph5*	ND	ARC10550	ND	ND	ND	4	[31]
Bph6*	4L	Swarnalata	Y19, Y9	21.36–21.39	Antibiosis, antixenosis	1, 2, 3, 4	[32,33]
Bph7*	12L	T12	RM3448, RM313	19.95–20.87	Antibiosis, tolerance	4	[27,34]
*bph8*	ND	Chin Saba	ND	ND	ND	1, 2, 3	[35]
Bph9*	12L	Pokkali	InD2, RsaI	22.85–22.97	Antibiosis, antixenosis	1, 2, 3	[36]
*Bph9-1*	12L	Kaharamana	RM463, RM5341	19.16–22.13	ND	1, 2, 3	[37]
*Bph10* *	12L	IR65482-4-136-2-2 (*O. australiensis*)	RG457	19.55–26.98	ND	1	[36,38]
*bph11*	3L	*O. officinalis*	G1318	35.60–35.80	ND	1, 2	[39]
*Bph12*	4S	B14 (*O. latifolia*)	RM16459, RM1305	5.21–5.56	Antibiosis, antixenosis	1, 2	[40,41]
*Bph13(t)-1*	2L	*O. eichingeri*	RM240, RM250	31.50–32.78	ND	ND	[42]
*Bph13(t)-2*	3S	*O.officinalis*	RG100, RG191	5.18–5.70	ND	4	[43]
*Bph14* *	3L	B5 (*O. officinalis*)	SM1, G1318	35.68~35.70	Antibiosis	1, 2, 3	[11]
Bph15*	4S	B5 (*O. officinalis*)	RG1, RG2	6.68~6.90	Antixenosis	1, 2, 3	[44,45]
*Bph16*	4L	*O. officinalis*	G271, R93	20.17~21.14	ND	1, 2	[39]
*Bph17*	4S	Rathu Heenati	RM8213, RM5953	4.44~9.38	ND	ND	[46]
*Bph18* *	12L	IR65482-7-216-1-2 (*O. australiensis*)	BIM3, BN162	22.88	Antibiosis, antixenosis	1	[36,47]
*bph18(t)*	4L	*O. rufipogon*	RM273, RM6506	24.05–25.05	ND	1, 2	[48]
*bph19(t)-1*	3S	AS20-1	RM6308, RM3134	7.18–7.24	ND	2	[49]
*bph19(t)-2*	12L	*O. rufipogon*	RM17	26.98	ND	1, 2	[48]
*Bph20(t)*	4S	IR71033-121-15 (*O. minuta*)	B42, B44	8.76	ND	1	[50]
*bph20(t)*	6S	*O. rufipogon*	BYL7, BYL8	0.47–0.53	ND	2	[51]
Bph21*	12L	IR71033-121-15 (*O. minuta*)	S12094A, B122	24.20–24.36	ND	1	[50]
*bph21(t)*	10S	*O. rufipogon*	RM222, RM244	2.62–5.00	ND	2	[51]
*Bph22(t)*	ND	*O. glaberrima*	ND	ND	ND	4	[52]
*Bph23(t)*	ND	*O. minuta*	ND	ND	ND	4	[52]
*bph22(t)*	4L	*O. rufipogon*	RM8212, RM261	19.11–19.57	ND	1, 2	[53]
*bph23(t)*	8L	*O. rufipogon*	RM2655, RM3572	16.63–17.07	ND	1, 2	[53]
*bph24(t)*	ND	IR73678-6-9-B (*O. rufipogon*)	ND	ND	ND	4	[54]
*Bph25*	6S	ADR52	S00310, RM8101	0.21	Antibiosis	ND	[55]
Bph26*	12L	ADR52	DS72B4, DS173B	22.87–22.89	Antibiosis	1, 2	[36,56]
*Bph27*	4L	GX2183 (*O. rufipogon*)	RM16846, RM16853	19.12–19.50	Antibiosis, antixenosis	1, 2	[57]
*Bph27(t)*	4L	Balamawee	Q52, Q20	20.79–21.33	Antibiosis, antixenosis	ND	[14]
*Bph28(t)*	11L	DV85	InDel55, InDel66	16.90–16.96	Tolerance	ND	[58]
Bph29*	6S	RBPH54 (*O. rufipogon*)	BYL8, BID2	0.48–0.49	ND	1, 2	[21]
Bph30*	4S	AC-1613	SSR28, SSR69	0.92–0.95	Antibiosis	1, 2, 3	[59]
*Bph31*	3L	CR2711-76	PA26, RM2334	26.26–26.74	Antibiosis, antixenosis, Tolerance	4	[60]
Bph32*	6S	Ptb33	RM19291, RM8072	1.21–1.40	Antibiosis	ND	[61]
*Bph33*	4S	KOLAYAL, PPLIYAL	H99, H101	0.91–0.97	Antibiosis, antixenosis	ND	[62]
*Bph33(t)*	1L	RP2068	RM488, RM11522	24.80–28.00	Antibiosis	ND	[63]
*Bph34*	4L	IRGC104646 (*O. nivara*)	RM16994, RM17007	21.32–21.47	ND	4	[64]
*Bph35*	4S	RBPH660 (*O. rufipogon*)	PSM16, RM413	6.28–6.94	ND	ND	[65]
*Bph36*	4S	GX2183 (*O. rufipogon*)	S13, X48	6.46–6.50	Antibiosis, antixenosis	1, 2	[66]
*Bph37-1*	1L	IR64	RM302, YM35	19.10–19.20	Tolerance	ND	[67]
Bph37-2*	6S	SE382	ND	1.20–1.50	ND	NDS	[68]
*Bph38*	4L	GX2183 (*O. rufipogon*)	YM112, YM190	15.00–15.10	Antibiosis, antixenosis	ND	[69]
*Bph38(t)*	1L	Khazar	SNP693369, id10112165	20.71–21.23	ND	3	[70]
*Bph39*	6S	Paedai Kalibungga	I7494, I1540	1.07–1.15	Antibiosis, antixenosis	ND	[71]
*bph39*	4S	RPBio4918-230S (*O. nivara*)	RM8213, RM5953	4.44–9.38	Antibiosis, tolerance	4S	[72]
*bph40*	4S	RPBio4918-230S (*O. nivara*)	RM5953, R4M17	9.38–11.4	Antibiosis, tolerance	4S	[72]
Bph40*	4S	SE232, SE67, C334	rs4_4486223	4.48–4.49	Antibiosis	1, 2, 3	[59]
*Bph41-1*	4S	GXU202(*O. rufipogon*)	W4_4_3, W1_6_3	4.68–4.78	ND	ND	[73]
*Bph41-2*	4S	SWD10	SWRm_01617, SWRm_01522	0.90–1.10	Antibiosis, antixenosis	4	[74]
*Bph42*	4L	SWD10	SWRm_01695, SWRm_00328	20.60–21.80	Antibiosis, antixenosis	4	[74]
*bph42*	4S	*O. rufipogon*	RM16282, RM16335	9.07–9.58	ND	4	[75]
*Bph43*	11L	IRGC 8678	InDel16_22, InDel16_30	16.79–16.90	Antibiosis, antixenosis	3	[76]
*Bph44*	4L	Balamawee	Q31, RM17007	21.38–21.47	Tolerance	1	[77]
*Bph45*	4L	TNG71(*O. nivara*)	RM16655, RM3317	13.70–13.80	Antixenosis	1	[10]
*Bph46*	12L	CL45	12M16.983, 12M19.042	16.99–19.04	Antibiosis	2	[78]
*qBph3*	3L	IR02W101 (O. *officinalis*)	t6, f3	35.47–35.63	ND	2	[79]
*qBph4*	4S	IR02W101 (*O. officinalis*)	P17, xc4_27	6.70–6.90	ND	2	[79]
*qBph6*	6S	IR71033-121-15	RM8120, RM8200	5.64–5.71	Antibiosis	ND	[80]
*qBph8*	8L	Swarnalata	RM339, RM515	17.94–20.28	Antixenosis	2	[81]
*qbph11*	11L	DV85	XNpb202, C1172	17.43–19.56	Tolerance	2	[82]
*qBph11.3*	11L	CL48	RM26567, 11MA104	16.80–16.90	Antibiosis	2	[78]
*qBph12*	12L	ASD7	RM3326, RM28597	21.80–24.70	Antibiosis	ND	[80]
*qBph4.1*	4S	Rathu Heenati	ND	5.78–7.78	ND	ND	[83]
*qBph4.2-1*	4S	IR65482-17 (*O. australiensis*)	RM261, XC4_27	6.58–6.89	Antibiosis	2	[84]
*qBph4.2-2*	4L	Rathu Heenati	ND	15.22–17.22	Antixenosis	ND	[83]
*qBph4.3*	4S	Salkathi	RM551, RM335	0.18~0.69	Antibiosis, antixenosis	4	[85]
*qBph4.4*	4S	Salkathi	RM335, RM5633	0.69~13.07	Antibiosis, antixenosis	4	[85]

Chr, chromosome; S, the short arm of chromosome; L, the long arm of chromosome; ND, no data. “*” means the genes have been cloned.

**Table 2:** BPH resistance genes cloned in rice.

Gene	Chr.	Germplasm	Encoded Protein	Subcellular Localization	Reference
*Bph1*	12L	Mudgo	CC-NB-NB-LRR	Endomembrane system	[36]
*Bph2*	12L	ASD7	CC-NB-NB-LRR	Endomembrane system	[36]
*Bph3*	4S	Rathu Heenati	LRK	Plasma membrane	[28]
*Bph6*	4L	Swarnalata	Atypical LRD	Exocyst	[32]
*Bph7*	12L	T12	CC-NB-NB-LRR	Endomembrane system	[36]
*Bph9*	12L	Pokkali	CC-NB-NB-LRR	Endomembrane system	[36]
*Bph10*	12L	IR65482-4-136-2-2 (O. *australiensis*)	CC-NB-NB-LRR	Endomembrane system	[36]
*Bph14*	3L	B5	CC-NB-LRR	Nucleus, cytoplasm	[11]
*Bph15*	4S	B5	LRK	Plasma membrane	[45]
*Bph18*	12L	IR65482-7-216-1-2 (O. *australiensis*)	CC-NB-NB-LRR	Endomembrane system	[47]
*Bph21*	12L	IR71033-121-15 (O. *minuta*)	CC-NB-NB-LRR	Endomembrane system	[36]
*Bph26*	6S	ADR52	CC-NB-NB-LRR	Endomembrane system	[36,56]
*Bph29*	6S	RBPH54	B3 DNA-binding	Nucleus	[21]
*Bph30*	4S	AC-1613	LRD	Endomembrane system	[59]
*Bph32*	6S	Ptb33	SCR	Plasma membrane	[61]
*Bph37*	6L	SE382	ND	CC-NB	[68]
*Bph40*	4S	SE232, SE67, C334	ND	LRD	[59]

Chr.: chromosome; S, the short arm of chromosome; L, the long arm of chromosome; CC-NB-LRR: coiled-coil nucleotide-binding leucine-rich repeat; LRK: lectin receptor kinase; LRD, leucine-rich repeat domain; SCR: short consensus repeat. ND, no data.

## Data Availability

All data have been included in the manuscript.

## References

[1] Yuan L. (2014). Development of Hybrid Rice to Ensure Food Security. Rice Sci..

[2] Bottrell D.G., Schoenly K.G. (2012). Resurrecting the ghost of green revolutions past: The brown planthopper as a recurring threat to high-yielding rice production in tropical Asia. J. Asia-Pac. Entomol..

[3] Du B., Chen R., Guo J., He G. (2020). Current understanding of the genomic, genetic, and molecular control of insect resistance in rice. Mol. Breed..

[4] Sōgawa K. (1982). The Rice Brown Planthopper: Feeding Physiology and Host Plant Interactions. Annu. Rev. Entomol..

[5] Min S., Lee S.W., Choi B.R., Lee S.H., Kwon D.H. (2014). Insecticide resistance monitoring and correlation analysis to select appropriate insecticides against *Nilaparvata lugens* (Stål), a migratory pest in Korea. J. Asia-Pac. Entomol..

[6] Wu S., Zeng B., Zheng C., Mu X., Zhang Y., Hu J., Zhang S., Gao C., Shen J. (2018). The evolution of insecticide resistance in the brown planthopper (*Nilaparvata lugens* Stål) of China in the period 2012–2016. Sci. Rep..

[7] Ling S., Zhang H., Zhang R. (2011). Effect of fenvalerate on the reproduction and fitness costs of the brown planthopper, *Nilaparvata lugens* and its resistance mechanism. Pestic. Biochem. Phys..

[8] Liu F., Li X., Zhao M., Guo M., Han K., Dong X., Zhao J., Cai W., Zhang Q., Hua H. (2020). Ultrabithorax is a key regulator for the dimorphism of wings, a main cause for the outbreak of planthoppers in rice. Nat. Sci. Rev..

[9] Khush, Gurdev S. (2001). Green revolution: The way forward. Nat. Rev. Genet..

[10] Li C., Wu D., Huang S., Meng M., Shih H., Lai M., Chen L., Jena K.K., Hechanova S.L., Ke T. (2023). The *Bph45* gene confers resistance against brown planthopper in rice by reducing the production of limonene. Int. J. Mol. Sci..

[11] Du B., Zhang W., Liu B., Hu J., Wei Z., Shi Z., He R., Zhu L., Chen R., Han B. (2009). Identification and characterization of *Bph14*, a gene conferring resistance to brown planthopper in rice. Proc. Natl. Acad. Sci. USA.

[12] Fujita D., Kohli A., Horgan F.G. (2013). Rice Resistance to Planthoppers and Leafhoppers. Crit. Rev. Plant Sci..

[13] Hao P., Liu C., Wang Y., Chen R., Tang M., Du B., Zhu L., He G. (2008). Herbivore-induced callose deposition on the sieve plates of rice: An important mechanism for host resistance. Plant Physiol..

[14] He J., Liu Y., Liu Y., Jiang L., Wu H., Kang H., Liu S., Chen L., Liu X., Cheng X. (2012). High-resolution mapping of brown planthopper (BPH) resistance gene *Bph27*(*t*) in rice (*Oryza sativa* L.). Mol. Breed..

[15] Painter R.H. (1958). Resistance of Plants to Insects. Annu. Rev. Entomol..

[16] Alam S.N., Cohen M.B. (1998). Detection and analysis of QTLs for resistance to the brown planthopper, *Nilaparvata lugens*, in a doubled-haploid rice population. Theor. Appl. Genet..

[17] Pathak M.D., Cheng C.H., Fortuno M.E. (1969). Resistance to *Nephotettix impicticeps* and *Nilaparvata lugens* in Varieties of Rice. Nature.

[18] Jackson M.T., Jackson M.T. (1997). Conservation of rice genetic resources: The role of the International Rice Genebank at IRRI. Plant Mol. Biol..

[19] Ling Y., Weilin Z. (2016). Genetic and biochemical mechanisms of rice resistance to planthopper. Plant Cell Rep..

[20] Ikeda R., Vaughan D. (1991). The distribution of resistance genes to the brown planthopper in rice germplasm. RGN.

[21] Wang Y., Cao L., Zhang Y., Cao C., Liu F., Huang F., Qiu Y., Li R., Lou X. (2015). Map-based cloning and characterization of *BPH29*, a B3 domain-containing recessive gene conferring brown planthopper resistance in rice. J. Exp. Bot..

[22] Athwal D. (1971). Genetics of resistance to brown planthoppers and green leafhoppers in *Oryza sativa* L.. Crop Sci..

[23] Kim S.M., Sohn J.K. (2005). Identification of a rice gene (*Bph1*) conferring resistance to brown planthopper (*Nilaparvata lugens* Stål) using STS markers. Mol. Cells.

[24] Sun L., Liu Y., Jiang L., Su C., Wang C., Zhai H., Wan J. (2007). Identification of quantitative trait loci associated with resistance to brown planthopper in the *indica* rice cultivar Col.5 Thailand. Hereditas.

[25] Sun L., Wang C., Su C., Liu Y., Zhai H., Wan J. (2006). Mapping and marker-assisted selection of a brown planthopper resistance gene *bph2* in rice (*Oryza sativa* L.). Acta Genet. Sin..

[26] Cha Y., Ji H., Yun D., Ahn B., Lee M.C., Suh S., Lee C.S., Ahn E.K., Jeon Y., Jin I. (2008). Fine mapping of the rice *Bph1* gene, which confers resistance to the brown planthopper (*Nilaparvata lugens* Stål), and development of STS markers for marker-assisted selection. Mol. Cells.

[27] Bhanu K.V., Lakshmi V.J., Katti G., Reddy A.V. (2014). Antibiosis and tolerance mechanisms of resistance in rice varieties carrying brown planthopper resistance genes. Asian J. Biol. Life Sci..

[28] Liu Y., Wu H., Chen H., Liu Y., He J., Kang H., Sun Z., Pan G., Wang Q., Hu J. (2014). A gene cluster encoding lectin receptor kinases confers broad-spectrum and durable insect resistance in rice. Nat. Biotechnol..

[29] Jairin J., Sansen K., Wongboon W., Kothcharerk J. (2010). Detection of a brown planthopper resistance gene bph4 at the same chromosomal position of *Bph3* using two different genetic backgrounds of rice. Breed. Sci..

[30] Sidhu G.S., Khush G.S. (1978). Genetic analysis of brown planthopper resistance in twenty varieties of rice, *Oryza saliva* L.. Theor. Appl. Genet..

[31] Khush G.S., Karim A.N.M.R., Angeles E.R. (1985). Genetics of resistance of rice cultivar ARC10550 to Bangladesh brown pianthopper teletype. J. Genet..

[32] Guo J., Xu C., Wu D., Zhao Y., Qiu Y., Wang X., Ouyang Y., Cai B., Liu X., Jing S. (2018). *Bph6* encodes an exocyst-localized protein and confers broad resistance to planthoppers in rice. Nat. Genet..

[33] Qiu Y., Guo J., Jing S., Zhu L., He G. (2010). High-resolution mapping of the brown planthopper resistance gene *Bph6* in rice and characterizing its resistance in the 9311 and Nipponbare near isogenic backgrounds. Theor. Appl. Genet..

[34] Qiu Y., Guo J., Jing S., Zhu L., He G. (2014). Fine mapping of the rice brown planthopper resistance gene *BPH7* and characterization of its resistance in the 93-11 background. Euphytica.

[35] Nemoto H., Ikeda R., Kaneda C. (1989). New genes for resistance to brown planthopper, *Nilaparvata lugens* Stål, in Rice. Jpn. J. Breed..

[36] Zhao Y., Huang J., Wang Z., Jing S., Wang Y., Ouyang Y., Cai B., Xin X.-F., Liu X., Zhang C. (2016). Allelic diversity in an NLR gene *BPH9* enables rice to combat planthopper variation. Proc. Natl. Acad. Sci. USA.

[37] Su C., Zhai H., Wang C., Sun L., Wan J. (2006). SSR mapping of brown planthopper resistance gene *Bph9* in kaharamana, an *indica* rice (*Oryza sativa* L.). Acta Genet. Sin..

[38] Ishii T., Brar D.S., Multani D.S., Khush G.S. (1994). Molecular tagging of genes for brown planthopper resistance and earliness introgressed from *Oryza australiensis* into cultivated rice, *O. sativa*. Genome.

[39] Hirabayashi H. (1998). Identification of brown planthopper resistance gene derived from *O. officinalis* using molecular markers in rice. Breed. Sci..

[40] Qiu Y., Guo J., Jing S., Zhu L., He G. (2011). Development and characterization of *japonica* rice lines carrying the brown planthopper-resistance genes *BPH12* and *BPH6*. Theor. Appl. Genet..

[41] Yang H., Ren X., Weng Q., Zhu L., He G. (2002). Molecular mapping and genetic analysis of a rice brown planthopper (*Nilaparvata lugens* Stål) resistance gene. Hereditas.

[42] Liu G., Yan H., Fu Q., Qian Q., Zhang Z., Zhai W., Zhu L. (2001). Mapping of a new gene for brown planthopper resistance in cultivated rice introgressed from *Oryza eichingeri*. Chin. Sci. Bull..

[43] Renganayaki K., Fritz A.K., Sadasivam S., Pammi S., Harrington S.E., McCouch S.R., Kumar S.M., Reddy A.S. (2002). Mapping and progress toward map-based cloning of brown planthopper biotype-4 resistance gene introgressed from *Oryza officinalis* into cultivated rice, *O. sativa*. Crop Sci..

[44] Yang H., You A., Yang Z., Zhang F., He R., Zhu L., He G. (2004). High-resolution genetic mapping at the *Bph15* locus for brown planthopper resistance in rice (*Oryza sativa* L.). Theor. Appl. Genet..

[45] Cheng X., Wu Y., Guo J., Du B., Chen R., Zhu L., He G. (2013). A rice lectin receptor-like kinase that is involved in innate immune responses also contributes to seed germination. Plant J..

[46] Sun L., Su C., Wang C., Zhai H., Wan J. (2005). Mapping of a major resistance gene to the brown planthopper in the rice cultivar Rathu Heenati. Breed. Sci..

[47] Ji H., Kim S., Kim Y.-H., Suh J.-P., Park H.-M., Sreenivasulu N., Misra G., Kim S.-M., Hechanova S.L., Kim H. (2016). Map-based cloning and characterization of the *BPH18* Gene from wild rice conferring resistance to brown planthopper (BPH) insect pest. Sci. Rep..

[48] Li R., Li L., Wei S., Wei Y., Chen Y., Bai D., Yang L., Huang F., Lu W., Zhang X. (2010). The evaluation and utilization of new genes for brown planthopper resistance in common wild rice (*Oryza rufipogon* Griff.). Mol. Entomol..

[49] Chen J.W., Wang L., Pang X.F., Pan Q.H. (2006). Genetic analysis and fine mapping of a rice brown planthopper (*Nilaparvata lugens* Stål) resistance gene *bph19*(*t*). Mol. Genet. Genom..

[50] Rahman M.L., Jiang W., Chu S.H., Qiao Y., Ham T., Woo M., Lee J., Khanam M.S., Chin J., Jeung J. (2009). High-resolution mapping of two rice brown planthopper resistance genes, *Bph20*(*t*) and *Bph21*(*t*), originating from *Oryza minuta*. Theor. Appl. Genet..

[51] Yang L., Li R.B., Li Y.R., Huang F.K., Chen Y.Z., Huang S.S., Huang L.F., Liu C., Ma Z.F., Huang D.H. (2011). Genetic mapping of *bph20*(*t*) and *bph21*(*t*) loci conferring brown planthopper resistance to *Nilaparvata lugens* Stål in rice (*Oryza sativa* L.). Euphytica.

[52] Ram T., Deen R., Gautam S., Ramesh K., Rao Y., Brar D. (2010). Identification of new genes for brown planthopper resistance in rice introgressed from *O. glaberrima* and *O. minuta*. Rice Genet. Newsl..

[53] Hou L., Yu P., Xu Q., Yuan X., Yu H., Wang Y., Wang C., Wan G., Tang S., Peng S. (2011). Genetic analysis and preliminary mapping of two recessive resistance genes to brown planthopper, *Nilaparvata lugens* Stål in rice. Rice Sci..

[54] Deen R., Ramesh K., Gautam S., Rao Y., Lakshmi V., Viraktamath B., Brar D., Ram T. (2010). Identification of new gene for BPH resistance introgressed from *O. rufipogon*. Rice Genet. Newsl..

[55] Myint K.K.M., Fujita D., Matsumura M., Sonoda T., Yoshimura A., Yasui H. (2011). Mapping and pyramiding of two major genes for resistance to the brown planthopper (*Nilaparvata lugens* Stål) in the rice cultivar ADR52. Theor. Appl. Genet..

[56] Tamura Y., Hattori M., Yoshioka H., Yoshioka M., Takahashi A., Wu J., Sentoku N., Yasui H. (2014). Map-based cloning and characterization of a brown planthopper resistance gene *BPH26* from *Oryza sativa* L. ssp. *indica* Cultivar ADR52. Sci. Rep..

[57] Huang D., Qiu Y., Zhang Y., Huang F., Meng J., Wei S., Li R., Chen B. (2013). Fine mapping and characterization of *BPH27*, a brown planthopper resistance gene from wild rice (*Oryza rufipogon* Griff.). Theor. Appl. Genet..

[58] Wu H., Liu Y., He J., Liu Y., Jiang L., Liu L., Wang C., Cheng X., Wan J. (2014). Fine mapping of brown planthopper (*Nilaparvata lugens* Stål) resistance gene *Bph28*(*t*) in rice (*Oryza sativa* L.). Mol. Breed..

[59] Shi S., Wang H., Nie L., Tan D., Zhou C., Zhang Q., Li Y., Du B., Guo J., Huang J. (2021). *Bph30* confers resistance to brown planthopper by fortifying sclerenchyma in rice leaf sheaths. Mol. Plant.

[60] Prahalada G.D., Shivakumar N., Lohithaswa H.C., Sidde Gowda D.K., Ramkumar G., Kim S.-R., Ramachandra C., Hittalmani S., Mohapatra T., Jena K.K. (2017). Identification and fine mapping of a new gene, *BPH31* conferring resistance to brown planthopper biotype 4 of India to improve rice, *Oryza sativa* L.. Rice.

[61] Ren J., Gao F., Wu X., Lu X., Zeng L., Lv J., Su X., Luo H., Ren G. (2016). *Bph32*, a novel gene encoding an unknown SCR domain-containing protein, confers resistance against the brown planthopper in rice. Sci. Rep..

[62] Hu J., Chang X., Zou L., Tang W., Wu W. (2018). Identification and fine mapping of *Bph33*, a new brown planthopper resistance gene in rice (*Oryza sativa* L.). Rice.

[63] Naik S.B., Divya D., Sahu N., Sundaram R.M., Sarao P.S., Singh K., Lakshmi V.J., Bentur J.S. (2018). A new gene *Bph33*(*t*) conferring resistance to brown planthopper (BPH), *Nilaparvata lugens* (Stål) in rice line RP2068-18-3-5. Euphytica.

[64] Kumar K., Sarao P.S., Bhatia D., Neelam K., Kaur A., Mangat G.S., Brar D.S., Singh K. (2018). High-resolution genetic mapping of a novel brown planthopper resistance locus, *Bph34* in *Oryza sativa* L. X *Oryza nivara* (Sharma & Shastry) derived interspecific F2 population. Theor. Appl. Genet..

[65] Zhang Y., Qin G., Ma Q., Wei M., Tang X., Ma Z., Liang H., Liu C., Li Z., Liu F. (2020). Identification of major locus *Bph35* resistance to brown planthopper in rice. Rice Sci..

[66] Li Z., Xue Y., Zhou H., Li Y., Usman B., Jiao X., Wang X., Liu F., Qin B., Li R. (2019). High-resolution mapping and breeding application of a novel brown planthopper resistance gene derived from wild rice (*Oryza. rufipogon* Griff). Rice.

[67] Yang M., Cheng L., Yan L., Shu W., Wang X., Qiu Y. (2019). Mapping and characterization of a quantitative trait locus resistance to the brown planthopper in the rice variety IR64. Hereditas.

[68] Zhou C., Zhang Q., Chen Y., Huang J., Guo Q., Li Y., Wang W., Qiu Y., Guan W., Zhang J. (2021). Balancing selection and wild gene pool contribute to resistance in global rice germplasm against planthopper. J. Integr. Plant Biol..

[69] Yang M., Lin J., Cheng L., Zhou H., Chen S., Liu F., Li R., Qiu Y. (2020). Identification of a novel planthopper resistance gene from wild rice (*Oryza rufipogon* Griff.). Crop J..

[70] Balachiranjeevi C.H., Prahalada G.D., Mahender A., Jamaloddin M., Sevilla M.A.L., Marfori-Nazarea C.M., Vinarao R., Sushanto U., Baehaki S.E., Li Z.K. (2019). Identification of a novel locus, *BPH38*(*t*), conferring resistance to brown planthopper (*Nilaparvata lugens* Stål.) using early backcross population in rice (*Oryza sativa* L.). Euphytica.

[71] Ye Y., Wang Y., Zou L., Wu X., Zhang F., Chen C., Xiong S., Liang B., Zhu Z., Wu W. (2024). Identification and candidate analysis of a new brown planthopper resistance locus in an Indian landrace of rice, paedai kalibungga. Mol. Breed..

[72] Srivastava A., Pusuluri M., Balakrishnan D., Vattikuti J.L., Neelamraju S., Sundaram R.M., Mangrauthia S.K., Ram T. (2023). Identification and functional characterization of two major loci associated with resistance against brown planthoppers (*Nilaparvata lugens* Stål) derived from *Oryza nivara*. Genes.

[73] Wang X., Han Y., Zhang Y., Deng B., Wu B., Guo X., Qin Y., Fang Y., Liu F., Qin B. (2022). QTL mapping integrated with BSA-Seq analysis identifies a novel gene conferring resistance to brown planthopper from common wild rice (*Oryza rufipogon* Griff.). Euphytica.

[74] Tan H.Q., Palyam S., Gouda J., Kumar P.P., Chellian S.K. (2022). Identification of two QTLs, *BPH41* and *BPH42*, and their respective gene candidates for brown planthopper resistance in rice. Sci. Rep..

[75] Kaur P., Neelam K., Sarao P.S., Babbar A., Kumar K., Vikal Y., Khanna R., Kaur R., Mangat G.S., Singh K. (2022). Molecular mapping and transfer of a novel brown planthopper resistance gene *bph42* from *Oryza rufipogon* (Griff.) to cultivated rice (*Oryza sativa* L.). Mol. Biol. Rep..

[76] Kim J., An X., Yang K., Miao S., Qin Y., Hu Y., Du B., Zhu L., He G., Chen R. (2022). Molecular mapping of a new brown planthopper resistance gene *Bph43* in rice (*Oryza sativa* L.). Agronomy.

[77] Kiswanto I., Soetopo L., Adiredjo A.L. (2022). Identification of novel candidate of brown planthopper resistance gene *Bph44* in rice (*Oryza sativa* L.). Genome.

[78] Li F., Yan L., Shen J., Liao S., Ren X., Cheng L., Li Y., Qiu Y. (2024). Fine mapping and breeding application of two brown planthopper resistance genes derived from landrace rice. PLoS ONE.

[79] Hu J., Xiao C., Cheng M., Gao G., Zhang Q., He Y. (2015). Fine mapping and pyramiding of brown planthopper resistance genes *QBph3* and *QBph4* in an introgression line from wild rice *O. officinalis*. Mol. Breed..

[80] Van Mai T., Fujita D., Matsumura M., Yoshimura A., Yasui H. (2015). Genetic basis of multiple resistance to the brown planthopper (*Nilaparvata lugens* Stål) and the green rice leafhopper (*Nephotettix cincticeps* Uhler) in the rice cultivar ‘ASD7’(*Oryza sativa* L. ssp. *indica*). Breed. Sci..

[81] Qiu Y., Cheng L., Liu F., Li R.B. (2013). Identification of a new locus conferring antixenosis to the brown planthopper in rice cultivar Swarnalata (*Oryza sativa* L.). Genet. Mol. Res..

[82] Su C., Wan J., Zhai H., Wang C., Sun L., Yasui H., Yoshimura A. (2005). A new locus for resistance to brown planthopper identified in the *indica* rice variety DV85. Plant Breed..

[83] Kamolsukyeunyong W., Ruengphayak S., Chumwong P., Kusumawati L., Chaichoompu E., Jamboonsri W., Saensuk C., Phoonsiri K., Toojinda T., Vanavichit A. (2019). Identification of spontaneous mutation for broad-spectrum brown planthopper resistance in a large, long-term fast neutron mutagenized rice population. Rice.

[84] Hu J., Xiao C., Cheng M., Gao G., Zhang Q., He Y. (2015). A new finely mapped *Oryza australiensis*-derived QTL in rice confers resistance to brown planthopper. Gene.

[85] Mohanty S.K., Panda R.S., Mohapatra S.L., Nanda A., Behera L., Jena M., Sahu R.K., Sahu S.C., Mohapatra T. (2017). Identification of novel quantitative trait loci associated with brown planthopper resistance in the rice landrace Salkathi. Euphytica.

[86] Hu L., Wu Y., Wu D., Rao W., Guo J., Ma Y., Wang Z., Shangguan X., Wang H., Xu C. (2017). The coiled-coil and nucleotide binding domains of BROWN PLANTHOPPER RESISTANCE14 function in signaling and resistance against planthopper in rice. Plant Cell.

[87] Guo J., Wang H., Guan W., Guo Q., Wang J., Yang J., Peng Y., Shan J., Gao M., Shi S. (2023). A tripartite rheostat controls self-regulated host plant resistance to insects. Nature.

[88] Hatchett J.H., Gallun R.L. (1970). Genetics of the ability of the hessian fly, mayetiola destructor, to survive on wheats having different genes for resistance. Ann. Entomol. Soc. Am..

[89] Laksminarayana A., Khush G.S. (1977). New genes for resistance to the brown planthopper in rice. Crop Sci..

[90] Cheng X., Zhu L., He G. (2013). Towards understanding of molecular interactions between rice and the brown planthopper. Mol. Plant.

[91] Jairin J., Phengrat K., Teangdeerith S., Vanavichit A., Toojinda T. (2007). Mapping of a broad-spectrum brown planthopper resistance gene, *Bph3*, on rice chromosome 6. Mol. Breed..

[92] Wu D., Guo J., Zhang Q., Shi S., Guan W., Zhou C., Chen R., Du B., Zhu L., He G. (2022). Necessity of rice resistance to planthoppers for OsEXO70H3 regulating SAMSL excretion and lignin deposition in cell walls. New Phytol..

[93] Li J., Liu X., Wang Q., Huangfu J., Schuman M.C., Lou Y. (2019). A group D MAPK protects plants from autotoxicity by suppressing herbivore-induced defense signaling. Plant Physiol..

[94] Ye W., Yu H., Jian Y., Zeng J., Ji R., Chen H., Lou Y. (2017). A salivary EF-hand calcium-binding protein of the brown planthopper *Nilaparvata lugens* functions as an effector for defense responses in rice. Sci. Rep..

[95] Dai Z., Tan J., Zhou C., Yang X., Yang F., Zhang S., Sun S., Miao X., Shi Z. (2019). The OsmiR396-OsGRF8-OsF3H-flavonoid pathway mediates resistance to the brown planthopper in rice (*Oryza sativa*). Plant Biotechnol. J..

[96] Liu M., Hong G., Li H., Bing X., Chen Y., Jing X., Gershenzon J., Lou Y., Baldwin I.T., Li R. (2023). Sakuranetin protects rice from brown planthopper attack by depleting its beneficial endosymbionts. Proc. Natl. Acad. Sci. USA.

[97] Sun B., Shen Y., Zhu L., Yang X., Liu X., Li D., Zhu M., Miao X., Shi Z. (2024). OsmiR319-OsPCF5 modulate resistance to brown planthopper in rice through association with MYB proteins. BMC Biol..

[98] He J., Liu Y., Yuan D., Duan M., Liu Y., Shen Z., Yang C., Qiu Z., Liu D., Wen P. (2019). An R2R3 MYB transcription factor confers brown planthopper resistance by regulating the phenylalanine ammonia-lyase pathway in rice. Proc. Natl. Acad. Sci. USA.

[99] Li S., Tan X., He Z., Jiang L., Li Y., Yang L., Hoffmann A.A., Zhao C., Fang J., Ji R. (2024). Transcriptome-wide N6-methyladenosine profiling reveals growth-defense trade-offs in the response of rice to brown planthopper (*Nilaparvata lugens*) infestation. Pest. Manag. Sci..

[100] Tao Z., Zhu L., Li H., Sun B., Liu X., Li D., Hu W., Wang S., Miao X., Shi Z. (2024). ACL1-ROC4/5 complex reveals a common mechanism in rice response to brown planthopper infestation and drought. Nat. Commun..

[101] Jiang Y., Zhang X., Li S., Xie Y., Luo X., Yang Y., Pu Z., Zhang L., Lu J., Huang H. (2024). Rapid intracellular acidification is a plant defense response countered by the brown planthopper. Curr. Biol..

[102] Qiu C.L., Li W., Wang L.N., Wang S.C., Falert S., Wang C., Yu S.Y., Abdelkhalek S.T., Lu J., Lin Y.J. (2024). Limonene enhances rice plant resistance to a piercing-sucking herbivore and rice pathogens. Plant Biotechnol. J..

[103] Pan G., Liu Y., Ji L., Zhang X., He J., Huang J., Qiu Z., Liu D., Sun Z., Xu T. (2018). Brassinosteroids mediate susceptibility to brown planthopper by integrating with the salicylic acid and jasmonic acid pathways in rice. J. Exp. Bot..

[104] Huangfu J., Li J., Li R., Ye M., Kuai P., Zhang T., Lou Y. (2016). The transcription factor OsWRKY45 negatively modulates the resistance of rice to the brown planthopper *Nilaparvata lugens*. Int. J. Mol. Sci..

[105] Bürger M., Chory J. (2019). Stressed out about hormones: How plants orchestrate immunity. Cell Host Microbe.

[106] Mishra A., Barik S.R., Pandit E., Yadav S.S., Das S.R., Pradhan S.K. (2022). Genetics, mechanisms and deployment of brown planthopper resistance genes in rice. Crit. Rev. Plant Sci..

[107] Zhou G., Qi J., Ren N., Cheng J., Erb M., Mao B., Lou Y. (2009). Silencing *OsHI-LOX* makes rice more susceptible to chewing herbivores, but enhances resistance to a phloem feeder. Plant J..

[108] Xu J., Wang X., Zu H., Zeng X., Baldwin I.T., Lou Y., Li R. (2021). Molecular dissection of rice phytohormone signaling involved in resistance to a piercing-sucking herbivore. New Phytol..

[109] Wang X., Chen Y., Liu S., Fu W., Zhuang Y., Xu J., Lou Y., Baldwin I.T., Li R. (2023). Functional dissection of rice jasmonate receptors involved in development and defense. New Phytol..

[110] Zhang X., Liu D., Gao D., Zhao W., Du H., Qiu Z., Huang J., Wen P., Wang Y., Li Q. (2022). Cytokinin confers brown planthopper resistance by elevating jasmonic acid pathway in rice. Int. J. Mol. Sci..

[111] Li M., Yu G., Cao C., Liu P. (2021). Metabolism, signaling, and transport of jasmonates. Plant Commun..

[112] Fu W., Jin G., Jiménez-Alemán G.H., Wang X., Song J., Li S., Lou Y., Li R. (2022). The jasmonic acid-amino acid conjugates JA-Val and JA-Leu are involved in rice resistance to herbivores. Plant Cell Environ..

[113] Dai Y.S., Liu D., Guo W., Liu Z.X., Zhang X., Shi L.L., Zhou D.M., Wang L.N., Kang K., Wang F.Z. (2023). Poaceae-specific β-1,3;1,4-d-glucans link jasmonate signalling to OsLecRK1-mediated defence response during rice-brown planthopper interactions. Plant Biotechnol. J..

[114] Lu J., Li J., Ju H., Liu X., Erb M., Wang X., Lou Y. (2014). Contrasting effects of ethylene biosynthesis on induced plant resistance against a chewing and a piercing-sucking herbivore in rice. Mol. Plant.

[115] Ma F., Yang X., Shi Z., Miao X. (2019). Novel crosstalk between ethylene- and jasmonic acid-pathway, *45*, responses to a piercing-sucking insect in rice. New Phytol..

[116] Huang J., Qiu Z., He J., Xu H., Wang K., Du H., Gao D., Zhao W., Sun Q., Wang Y. (2023). Phytochrome B mediates dim-light-reduced insect resistance by promoting the ethylene pathway in rice. Plant Physiol..

[117] Zhou Y., Sun L., Wang S., Xie P., Liu J. (2019). A key ABA hydrolase gene, OsABA8ox3 is involved in rice resistance to *Nilaparvata lugens* by affecting callose deposition. J. Asia-Pac. Entomol..

[118] Li J., Liu H., Lv X., Wang W., Liang X., Chen L., Wang Y., Liu J. (2024). A key ABA biosynthetic gene *OsNCED3* is a positive regulator in resistance to *Nilaparvata lugens* in *Oryza sativa*. Front. Plant Sci..

[119] Liu J., Du H., Ding X., Zhou Y., Xie P., Wu J. (2017). Mechanisms of callose deposition in rice regulated by exogenous abscisic acid and its involvement in rice resistance to *Nilaparvata lugens* Stål (Hemiptera: Delphacidae). Pest. Manag. Sci..

[120] Li R., Zhang J., Li J., Zhou G., Wang Q., Bian W., Erb M., Lou Y. (2015). Prioritizing plant defence over growth through WRKY regulation facilitates infestation by non-target herbivores. eLife.

[121] Zhang J., Luo T., Wang W., Cao T., Li R., Lou Y. (2017). Silencing *OsSLR1* enhances the resistance of rice to the brown planthopper *Nilaparvata lugens*. Plant Cell Environ..

[122] Chen L., Cao T., Zhang J., Lou Y. (2018). Overexpression of *OsGID1* enhances the resistance of rice to the brown planthopper *Nilaparvata lugens*. Int. J. Mol. Sci..

[123] Wang W., Jin N., Mo X., Wu J., Lu J., Lou Y. (2020). Exogenous gibberellin GA3 enhances defense responses in rice to the brown planthopper *Nilaparvata lugens* (Stål). J. Plant Biol..

[124] Zhang Q., Li T., Gao M., Ye M., Lin M., Wu D., Guo J., Guan W., Wang J., Yang K. (2022). Transcriptome and metabolome profiling reveal the resistance mechanisms of rice against brown planthopper. Int. J. Mol. Sci..

[125] Shi S., Zha W., Yu X., Wu Y., Li S., Xu H., Li P., Li C., Liu K., Chen J. (2023). Integrated transcriptomics and metabolomics analysis provide insight into the resistance response of rice against brown planthopper. Front. Plant Sci..

[126] Huang S., Jia A., Ma S., Sun Y., Chang X., Han Z., Chai J. (2023). NLR signaling in plants: From resistosomes to second messengers. Trends Biochem. Sci..

[127] Kuai P., Lin N., Ye M., Ye M., Chen L., Chen S., Zu H., Hu L., Gatehouse A.M.R., Lou Y. (2024). Identification and knockout of a herbivore susceptibility gene enhances planthopper resistance and increases rice yield. Nat. Food.

[128] McDowell J.M., Argueso C.T., Ferreira F.J., Epple P., To J.P.C., Hutchison C.E., Schaller G.E., Dangl J.L., Kieber J.J. (2012). Two-component elements mediate interactions between cytokinin and salicylic acid in plant immunity. PLoS Genet..

[129] Cao M., Chen R., Li P., Yu Y., Zheng R., Ge D., Zheng W., Wang X., Gu Y., Gelová Z. (2019). TMK1-mediated auxin signalling regulates differential growth of the apical hook. Nature.

[130] Gao J., Chen H., Yang H., He Y., Tian Z., Li J. (2018). A brassinosteroid responsive miRNA-target module regulates gibberellin biosynthesis and plant development. New Phytol..

[131] Shen Y., Yang G., Miao X., Shi Z. (2023). OsmiR159 modulate bph resistance through regulating g-protein γ subunit *GS3* gene in rice. Rice.

[132] Xiao L., Gheysen G., Yang M., Xiao X., Xu L., Guo X., Yang L., Liu W., He Y., Peng D. (2024). Brown planthopper infestation on rice reduces plant susceptibility to *Meloidogyne graminicola* by reducing root sugar allocation. New Phytol..

[133] Lozano-Durán R., Zipfel C. (2015). Trade-off between growth and immunity: Role of brassinosteroids. Trends Plant Sci..

[134] Jin G., Qi J., Zu H., Liu S., Gershenzon J., Lou Y., Baldwin I.T., Li R. (2023). Jasmonate-mediated gibberellin catabolism constrains growth during herbivore attack in rice. Plant Cell.

[135] Ning Y., Liu W., Wang G.-L. (2017). Balancing immunity and yield in crop plants. Trends Plant Sci..

[136] Nomoto M., Skelly M.J., Itaya T., Mori T., Suzuki T., Matsushita T., Tokizawa M., Kuwata K., Mori H., Yamamoto Y.Y. (2021). Suppression of MYC transcription activators by the immune cofactor NPR1 fine-tunes plant immune responses. Cell Rep..

[137] Liu Y., Chen L., Liu Y., Dai H., He J., Kang H., Pan G., Huang J., Qiu Z., Wang Q. (2016). Marker assisted pyramiding of two brown planthopper resistance genes, *Bph3* and *Bph27*(*t*), into elite rice Cultivars. Rice.

[138] Liu M., Fan F., He S., Guo Y., Chen G., Li N., Li N., Yuan H., Si F., Yang F. (2022). Creation of elite rice with high-yield, superior-quality and high resistance to brown planthopper based on molecular design. Rice.

[139] van Schie C.C., Takken F.L. (2014). Susceptibility genes 101: How to be a good host. Annu. Rev. Phytopathol..

[140] Gao M., He Y., Yin X., Zhong X., Yan B., Wu Y., Chen J., Li X., Zhai K., Huang Y. (2021). Ca^2+^ sensor-mediated ROS scavenging suppresses rice immunity and is exploited by a fungal effector. Cell.

[141] Li S., Lin D., Zhang Y., Deng M., Chen Y., Lv B., Li B., Lei Y., Wang Y., Zhao L. (2022). Genome-edited powdery mildew resistance in wheat without growth penalties. Nature.

[142] Yu L., Chen Y., Zeng X., Lou Y., Baldwin I.T., Li R. (2024). Brown planthoppers manipulate rice sugar transporters to benefit their own feeding. Curr. Biol..

[143] Jones A.C., Felton G.W., Tumlinson J.H. (2022). The dual function of elicitors and effectors from insects: Reviewing the ‘arms race’ against plant defenses. Plant Mol. Biol..

